# Associations between Different Ozone Indicators and Cardiovascular Hospital Admission: A Time-Stratified Case-Crossover Analysis in Guangzhou, China

**DOI:** 10.3390/ijerph20032056

**Published:** 2023-01-23

**Authors:** Xiangxue Zhang, Kamal Jyoti Maji, Zhuoqing Wang, Fiona Fan Yang, Guobin Wang, Changxiu Cheng

**Affiliations:** 1 State Key Laboratory of Earth Surface Processes and Resource Ecology, Beijing Normal University, Beijing 100875, China; 2 Faculty of Geo-Information Science and Earth Observation (ITC), University of Twente, 7514 AE Enschede, The Netherlands; 3 School of Civil and Environmental Engineering, Georgia Institute of Technology, Atlanta, GA 30332, USA; 4 Department of Scientific Research & Discipline Development, The First Affiliated Hospital, Sun Yat-Sen University, Guangzhou 510080, China; 5 School of Geography and Planning, Sun Yat-Sen University, Guangzhou 510006, China; 6 National Tibetan Plateau Data Center, Beijing 100101, China

**Keywords:** cardiovascular diseases, hospital admissions, ozone, time-stratified case crossover

## Abstract

Epidemiological studies reported that ozone (O_3_) is associated with cardiovascular diseases. However, only few of these studies examined the impact of multiple O_3_ indicators on cardiovascular hospital admissions. This study aimed to explore and compare the impacts of different O_3_ indicators on cardiovascular hospital admissions in Guangzhou, China. Based upon the data on daily cardiovascular hospital admissions, air pollution, and meteorological factors in Guangzhou from 2014 to 2018, a time-stratified case-crossover design model was used to analyze the associations between different O_3_ indicators and cardiovascular hospital admissions. Moreover, the sensitivities of different age and gender groups were analyzed for the whole year and different seasons (i.e., warm and cold). During the warm season, for the single-pollutant model, the odds ratio (OR) value of cardiovascular hospital admissions was 1.0067 (95% confidence interval (CI): 1.0037, 1.0098) for every IQR increase in MDA8 O_3_ at a lag of five days. The effect of O_3_ on people over 60 year was stronger than that on the 15–60 years age group. Females were more sensitive than males to O_3_ exposure. These results provided valuable references for further scientific research and environmental improvement in Guangzhou. Given that short-term O_3_ exposure poses a threat to human health, the government should therefore pay attention to prevention and control policies to reduce and eliminate O_3_ pollution and protect human health.

## 1. Introduction

Ozone (O_3_), a secondary pollutant, is formed by the photochemical reactions of precursors that mainly include volatile organic compounds (VOCs) and nitrogen oxides (NOx). It can negatively impact human and animal health, plant growth, climate change, and ecological balance on a local, regional, or even global scale [1,2,3,4]. O_3_ pollution poses a huge threat to the global public health, especially in developing countries such as China and India [5,6]. In recent years, rapid economic development and urbanization have been closely related to severe O_3_ pollution. Although China implemented an important policy in 2013 (i.e., Air Pollution Prevention and Control Action Plan), the concentration of particulate matter dominated by PM_2.5_ (particulate matter with an aerodynamic diameter ≤2.5 μm) shows an obvious decreasing trend, while O_3_ concentration shows an increasing trend, gradually becoming the dominant air pollutant in China [7,8]. Notably, the reduction of PM_2.5_ slows down the absorption of hydroperoxyl radicals, thereby accelerating the formation of O_3_ [9]. As China is facing a serious O_3_ pollution problem, it is therefore necessary to fully understand the susceptible diseases associated with O_3_ exposure.

O_3_ exposure not only has a negative impact on human health [10], but also imposes great economic burden [6,11,12]. Epidemiological studies showed that short-term O_3_ exposure leads to platelet activation and increases blood pressure and is positively associated with cardiovascular mortality [2,13,14,15]. Moreover, a high O_3_ concentration can cause respiratory diseases and aggravate the conditions of patients with such diseases, including asthma and chronic lung disease [16]. Therefore, examining the impact of O_3_ on human health, especially in the period of severe O_3_ pollution, is an urgent concern.

Many epidemiological studies have evaluated the impact of O_3_ pollution on human health worldwide. It is worth noting that the selection of O_3_ indicators will affect the results of risk estimation [17,18,19]. At present, three O_3_ indicators, which represent different exposure times are commonly used in epidemiological studies; the maximum daily 8 h average O_3_ concentration (MDA8 O_3_), the maximum daily 1 h average O_3_ concentration (MDA1 O_3_), and the daily 24 h average O_3_ concentration are also considered [17,20]. For example, Yang et al. (2004) used a case-crossover method to estimate whether an association exists between O_3_ concentration (daily 24 h average O_3_ concentration) and cardiovascular hospital admissions in Taiwan [21]. Their results showed that O_3_ was significantly and positively associated with cardiovascular hospital admissions in the warm season. On the basis of a generalized additive model, Wu et al. (2022) investigated the health effects of O_3_ exposure (MDA8 O_3_) on cardiovascular mortality in Nanchang, China [2]. Their results showed that, for every 10 μg/m^3^ rise in O_3_ concentration, cardiovascular mortality increased by 1.26% (95% confidence interval (CI): 0.68, 1.83%). Using a generalized additive model as well, Yang et al. (2012) estimated the effect of O_3_ concentration (MDA1 O_3_) on cardiovascular mortality from 2006 to 2008 in Suzhou, China [19]. Their results showed that O_3_ level was positively associated with cardiovascular mortality, with every 10 μg/m^3^ increase in MDA1 O_3_ related to a 4.31% (95% CI: 1.34, 7.27%) increase in cardiovascular mortality.

Most of the previous studies only included a single O_3_ indicator, and few analyzed different O_3_ indicators simultaneously and compared their associations with cardiovascular admissions. Accordingly, this study aimed to (1) use a time-stratified case-crossover (TSCC) model to explore and identify the short-term impact of different O_3_ indicators (MDA8 O_3_ and MDA1 O_3_) on daily cardiovascular hospital admissions in Guangzhou, China; (2) investigate the association between different O_3_ indicators and daily cardiovascular hospital admissions in different age and gender groups for the whole year and for different seasons (warm and cold); and (3) compare the results of the single-day lag model and the moving average lag model, as well as perform further sensitivity analysis.

## 2. Method and Data Sources

### 2.1. Hospital Admissions

Guangzhou is the largest metropolis in South China, with more than 18 million people. Owing to rapid economic development and increased energy consumption in the past few decades, Guangzhou has suffered severe air pollution, possibly caused by the considerable emissions of anthropogenic precursors [22]. In 2019, the annual average O_3_ concentration in Guangzhou was 178 μg/m^3^, far more than the standard (100 μg/m^3^) recommended by the World Health Organization (WHO) [23]. The dense population and high O_3_ concentration in Guangzhou contributed to its selection as a typical city to examine the health effects of O_3_ pollution. In this study, data on daily cardiovascular hospital admissions from 1 January 2014 to 31 August 2018 collected from the First Affiliated Hospital of Sun Yat-Sen University, located in Yuexiu District, central Guangzhou, were used to investigate the association between O_3_ and cardiovascular hospital admissions. The data information included: hospitalization date, gender, age, residential address, and diagnosis from the 10^th^ International Classification of Diseases (ICD-10: I01-I59). As 93.65% of the people in this dataset were over 15 years old, the hospital admissions data in this study were thus divided into two age groups: 15–60 and 60+ years old. Screening was performed according to the residential address of the patients, who were all residents of Guangzhou.

### 2.2. Air Pollutants and Meteorological Data

The data on pollutants from January 1, 2014 to August 31, 2018, including O_3_ (MDA8 O_3_ and MDA1 O_3_), PM_2.5_, and NO_2_, were obtained from the Guangzhou Air Quality Monitoring Station. Among them, PM_2.5_ and NO_2_ concentrations were used to identify the stability of the relationship between O_3_ and cardiovascular admissions in the multi-pollution model. The pollutant data were collected from the National Environmental Monitoring Center of China (http://quotsoft.net/air/ accessed on 8 July 2022). The 10 air quality monitoring stations in Guangzhou can be divided into three categories, namely, urban, suburban, and mountainous stations, which are located in the densely populated area with high traffic pressure, areas where the industrial base is less developed and the population is relatively sparse, and areas that have high altitude and location in mountainous scenic spots. More importantly, due to data maybe being affected by unpredictable weather conditions, equipment/data transmission failures, etc., the O_3_ monitoring data contain a large number of missing values and potential outliers. To guarantee the stability of the results, we adopt a strict quality control of the data according to [24]. For each city, when we have not less than 20 h of data, then the daily O_3_ concentration was the average of the hourly data of all monitoring stations in this city [8]. Moreover, if a large amount of data was lost on a day, then the data for that day were considered as invalid and replaced with the average value from the corresponding month [25]. Considering that the hospitalized patients came from different districts in Guangzhou, the average value of pollutants from 10 monitoring stations was thus used in this study.

The meteorological data from 2014 to 2018 were obtained from the China Meteorological Data Sharing Service System (http://data.cma.gov.cn/). Daily average temperature and relative humidity were included to test the sensitivity of the results. All the data were divided into two seasons, warm and cold, according to their monthly average temperature. In particular, months with an average temperature above 20 °C were classified as warm season (May to October) and those below 20 °C were classified as cool season (November to April).

### 2.3. Statistical Analysis

Given that each case serves as its own control, a TSCC design can well control the temporal and seasonal confounders, as well as individual confounders, such as age, gender, and socioeconomic status. Therefore, TSCC is widely used in epidemiological studies [26,27]. Time-stratified refers to the method of comparing the day a patient was hospitalized (case day) with the same day in other weeks within one previous month (control day) [28]. Specifically, if a patient visited on a Tuesday in August 2015, all Tuesdays a month prior would be control days; according to this rule, every case will thus have three to four controls.

In the present study, TSCC was used to evaluate the effect of short-term exposure to different O_3_ indicators on cardiovascular hospital admissions. We used conditional logistic regression to generate the odds ratio (OR) values and their 95% CI. If the value of *p* was less than 0.05, the results were considered statistically significant. Additionally, daily average temperature and relative humidity were involved as covariates. The model is as follows:

(1) $$\log\;\left(h\left(t,X\right)\right)=\log\;\left(h_{0}\left(t\right)\right)+C_{s}\beta_{1}+Temp\beta_{2}+RH\beta_{3}$$

where *t* is the date of hospital admission, and *X* represents the independent variable, including pollutants and meteorological factors. The item *log* (*h* (*t, X*)) represents the risk function of exposure to *X* on the day *t*, and *log* (*h_0_* (*t*)) refers to the baseline risk function [28]. *C_s_* is the daily concentration of pollutants; and *Temp* and *RH* represent average temperature and relative humidity, respectively, with the coefficients *β_1_*, *β_2_*, and *β_3_*.

To examine whether O_3_ has a lag effect on cardiovascular hospital admissions, we analyzed the impacts of O_3_ with a single-day lag model (from lag0 to lag5) and a multi-day lag model (from lag01 to lag05). The pollutant concentration of lag0 refers to the concentration on the current day, and the pollutant concentration of lag1 refers to the O_3_ concentration of the previous one day. The pollutant concentration of lag01 represents the 2-day average concentration of the pollutant concentrations on the current day and the previous one day, and the pollutant concentration of lag05 represents the concentration averaged on the current day and the previous five days. In the lag model, we obtained the ORs and optimal lag period for O_3_ based on the maximum value of the ORs.

We also performed a series of subgroup analyses stratified by age (15–60 years age group and more than 60 years age group) and gender (male and female) to identify potentially susceptible subgroups. These age stratifications refer to previously published studies [5,29]. Seasonal analysis of O_3_-related effects was also performed by dividing the whole year into warm season (May to October) and cold season (November to April). The effects of different O_3_ indicators on daily cardiovascular hospital admissions in Guangzhou were both examined in these subgroup analyses.

### 2.4. Sensitivity Analysis

To further verify the stability of the results, we (1) performed a multi-pollutant analysis, where we added other severe pollutants (e.g., NO_2_ and PM_2.5_) one by one to test whether the results were stable; and (2) we changed the lag days of meteorological factors (i.e., average temperature and relative humidity) from lag0 to lag3 to test whether the results were sensitive to changes in meteorological factors.

All of the above analyses were performed in R software (version 3.6.3). The *p*-values less than 0.05 were regarded as statistically significant.

## 3. Results

### 3.1. Basic Information of Data

Table 1 describes the basic statistical characteristics of the daily data on cardiovascular hospital admissions, two different O_3_ indicators, two other major air pollutants (PM_2.5_ and NO_2_), and meteorological factors (average temperature and relative humidity). From 1 January 2014 to 31 August 2018, the cumulative number of inpatients with cardiovascular disease was 20,356, of which 11,360 (55.8%) were male and 8996 (44.2%) were female. There were 13,936 (68.5%) people aged 60+ years old and 6420 (31.5%) people aged 15–60 years old. The average daily concentrations of MDA8 O_3_, MDA1 O_3_, PM_2.5_, and NO_2_ were 91 ± 51, 105 ± 59, 38 ± 22, and 46 ± 18 μg/m^3^, respectively.

**Figure 1:**
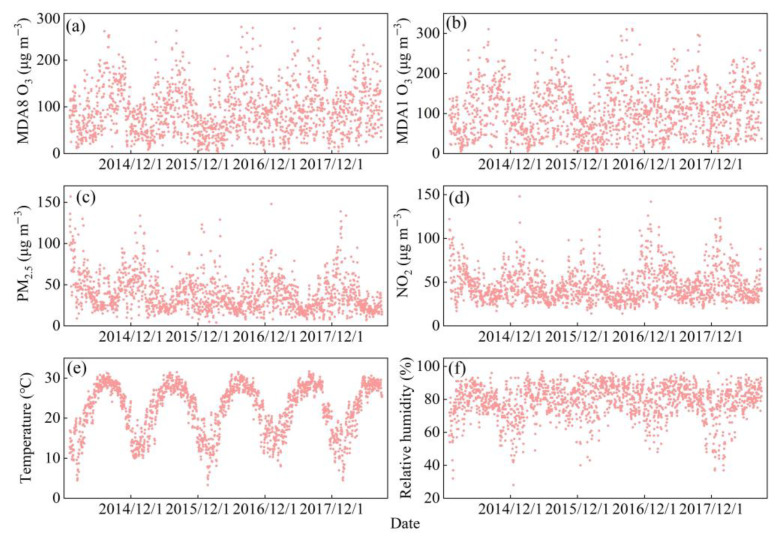
Time series of monthly average air pollutants and meteorological factors in Guangzhou, China from 2014 to 2018.

Notably, PM_2.5_ and NO_2_ concentrations were higher in the cold season, whereas O_3_ was higher in the warm season. The daily concentration range of MDA8 O_3_ and MDA1 O_3_ during the study period (2014–2018) were 4.0–271.0 and 4.3–311.1 μg/m^3^, respectively. The daily concentration of NO_2_ was in the range of 14.0–148.0 μg/m^3^, with an annual average of 46.1 μg/m^3^. During the study period, NO_2_ exceeded the level II of the Chinese Ambient Air Quality Standards (40 μg/m^3^) for 963 days, accounting for 54.93% of the total number of days. The annual average value of PM_2.5_ was 38.2 μg/m^3^, which was 46.8% higher than the 35 μg/m^3^ threshold recommended by the level II of the Chinese Ambient Air Quality Standards, and it was 99.0% higher than the annual average reported by the WHO (10 μg/m^3^). Moreover, the daily average temperature and relative humidity ranged from 3.3 °C to 31.7 °C (annual average was 22.2 °C) and from 28% to 97% (annual average was 78.7%), respectively, which were included in the model as confounding factors. Figure 1 shows the temporal evolution of the four pollutants and the two meteorological factors.

### 3.2. The Relationship between Ozone and Cardiovascular Hospitalization

**Figure 2:**
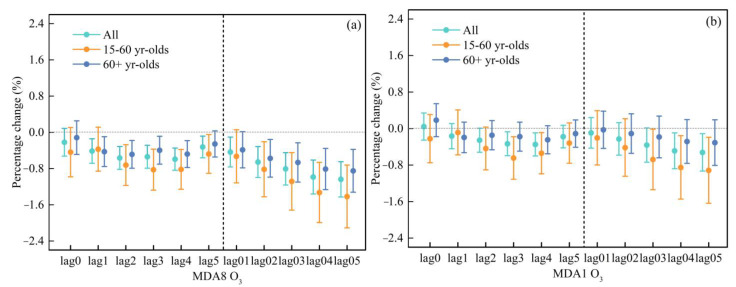
Comparison the effects for an IQR increase in different O_3_ indicators (MDA8 O_3_ (**a**) and MDA1 O_3_ (**b**)) on cardiovascular hospitalization admissions in different age groups.

Figure 2 shows the percentage change in cardiovascular hospital admissions with an IQR increase in MDA8 O_3_ and MDA1 O_3_. Figure S1 shows the association between two O_3_ indicators and the daily cardiovascular hospital admissions in a single pollutant model. Similar lagged effects were observed for the two O_3_ indicators. For MDA8 O_3_, significant and negative OR values were observed at lag1-lag05, and only the result of lag0 was not statistically significant (Figure S1a). For MDA1 O_3_, significant and negative OR values of 0.9966 (95% CI: 0.9940, 0.9993), 0.9965 (95% CI: 0.9940, 0.9990), 0.9951 (95% CI: 0.9912, 0.9990), and 0.9947 (95% CI: 0.9907, 0.9989) were observed at lag3, lag4, lag04, and lag05 days, respectively (Figure S1b).

The risk of cardiovascular hospital admissions in different age groups was further examined by applying the same model to determine whether there were differences between age groups. As shown in Figure S1a, MDA8 O_3_ was negatively related to cardiovascular hospital admissions. At the lag of five days, the effect of MDA8 O_3_ on cardiovascular hospital admissions in the 15–60 years age group was the highest and significant, with an OR value of 0.9999 (95% CI: 0.9952, 0.9909). The highest OR value at the day of lag3 was significant for the 60+ years age group, with an OR value of 0.9960 (95% CI: 0.9929, 0.9991).

For MDA1 O_3_, its effect on the 15–60 years age group was significant and the highest at the day of lag4, with an OR value of 0.9946 (95% CI: 0.9901, 0.9991), while its effect on the 60+ years age group had no significant association (Figure S1b).

The OR estimates for different gender groups were further analyzed using the same model (Figure S2). For MDA8 O_3_, at the lag of three days, the effect of MDA8 O_3_ on cardiovascular hospital admissions in the male group was the highest and significant, with an OR value of 0.9965 (95% CI: 0.9931, 0.9998). For the female group, the highest and significant association occurred at the day of lag 2, with an OR value of 0.9951 (95% CI: 0.9913, 0.9989) (Figure S2a).

**Figure 3:**
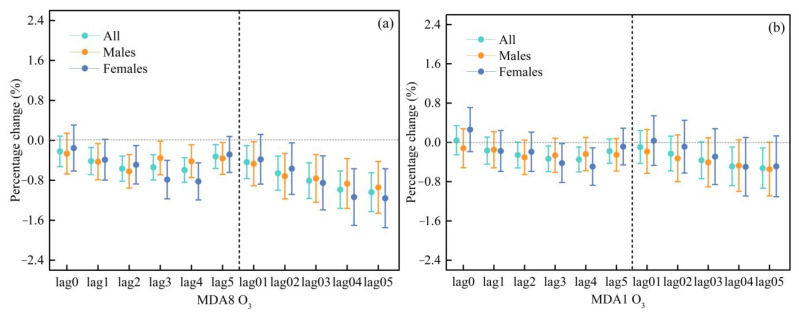
Comparison the effects for an IQR increase in different O_3_ indicators (MDA8 O_3_ (**a**) and MDA1 O_3_ (**b**)) on cardiovascular hospitalization admissions in different gender groups.

For MDA1 O_3_, no significant association was found for the male group. Meanwhile, for the female group, significant associations were found at the days of lag3 and lag4, with OR values of 0.9958 (95% CI: 0.9918, 0.9997) and 0.9951 (95% CI: 0.9913, 0.9989), respectively (Figure S2b). Figure 3 shows the percentage change in cardiovascular hospital admissions with an IQR increase in MDA8 O_3_ and MDA1 O_3_ for different gender people.

### 3.3. Seasonal Estimates of the Effects of Different Ozone Indicators on Cardiovascular Hospital Admissions

**Figure 4:**
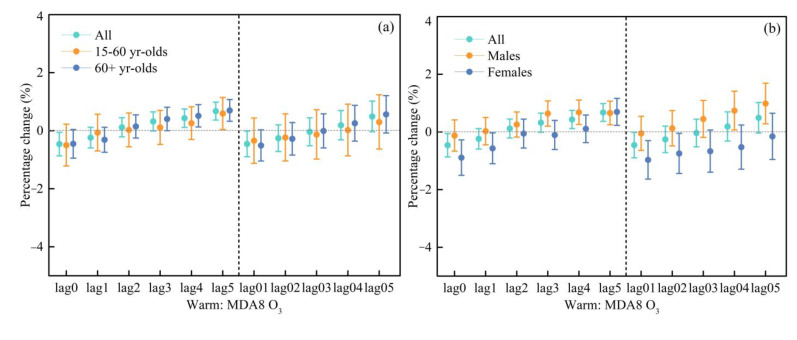

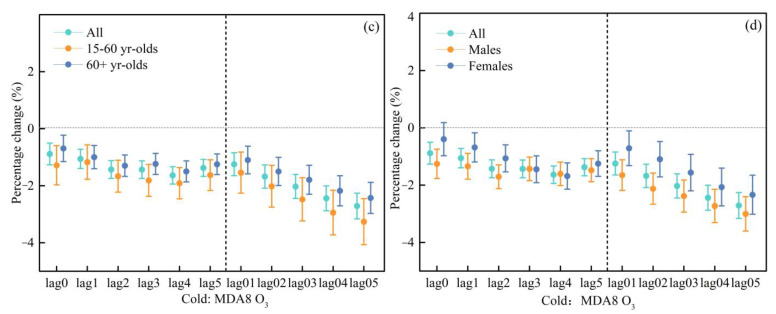
Comparison of the effects for an IQR increase in MDA8 O_3_ on cardiovascular hospitalization admissions during the warm periods (**a**,**b**) and the cold periods (**c**,**d**).

**Figure 5:**
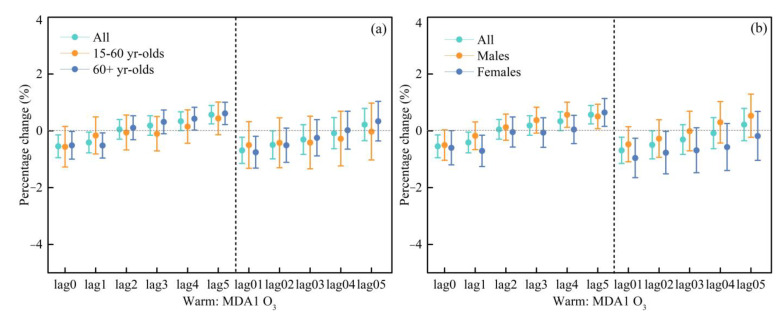

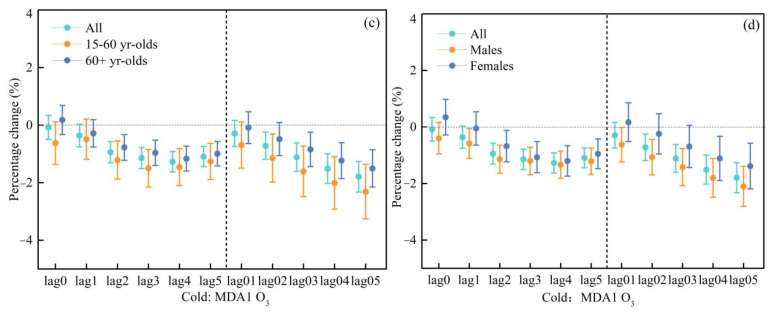
Comparison the effects for an IQR increase in MDA1 O_3_ on cardiovascular hospitalization admissions during the warm periods (**a**,**b**) and the cold periods (**c**,**d**).

The continuous high temperature and strong sunshine in the warm season are conducive to the photochemical reaction of VOCs and NOx, which will accelerate the formation of O_3_. That is, there is a phenomenon that the O_3_ concentration in summer is higher than that in winter. Therefore, we evaluated the effect of different O_3_ indicators on cardiovascular hospital admissions in the warm season (May to October) and the cold season (November to April) (Figures S3 and S4, respectively). The results showed that O_3_ had different effects on cardiovascular hospital admissions in different seasons. Specifically, a significant and positive association between MDA8 O_3_/MDA1 O_3_ and cardiovascular hospital admissions in the warm season and a negative association in the cold season. Figure 4 and Figure 5 show the percentage change in cardiovascular hospital admissions with an IQR increase in MDA8 O_3_ and MDA1 O_3_ in different seasons.

During the warm season, similar lagged effects on cardiovascular hospital admissions were observed for the two different O_3_ indicators. MDA8 O_3_ was significantly and positively related to all the people from lag4 and lag5 days, with OR values of 1.0043 (95% CI: 1.0011, 1.0075) and 1.0067 (95% CI: 1.0037, 1.0098), respectively (Figure S3). For the moving average lag model, the results were not significant. As for MDA1 O_3_, there was also a positive association at a lag of four and five days, with the OR values of 1.0034 (95% CI: 1.0001, 1.0067) and 1.0057 (95% CI: 1.0024, 1.0089), respectively (Figure S4). In the cold season, MDA8 O_3_/MDA1 O_3_ was significantly and negatively correlated with cardiovascular hospital admissions (Figure S3 and S4).

We further examined the OR value in different seasons by using the same model to determine whether there were differences between different seasons. As shown in Figure 4, MDA8 O_3_ was positively associated with the cardiovascular hospital admissions in the warm periods. At the lag of five days, the effect of MDA8 O_3_ on cardiovascular hospital admissions in the 15–60 years age group was the highest and significant, with an OR value of 1.0059 (95% CI: 1.0004, 1.0115). Meanwhile, significant and positive associations for the 60+ years age group were found at the days of lag3 to lag5, with OR values of 1.0040 (95% CI: 1.0000, 1.0081), 1.0051 (95% CI: 1.0013, 1.0090), and 1.0070 (95% CI: 1.0033, 1.0107), respectively (Figure S3a).

For MDA1 O_3_, its significant effect on the 60+ years age group was observed at the days of lag4 and lag5, with OR values of 1.0043 (95% CI: 1.0002, 1.0083) and 1.0061 (95% CI: 1.0022, 1.0101), respectively. Meanwhile, its effect on the 15–60 years age group had no significant association (Figure S4a).

For MDA8 O_3_, significant associations were found for the male group at the days of lag3 to lag5, with OR values of 1.0064 (95% CI: 1.0020, 1.0108), 1.0068 (95% CI: 1.0026, 1.0110), and 1.0066 (95% CI: 1.0025, 1.0107), respectively. Meanwhile, for the female group, the significant and highest association was found at the day of lag5, with an OR value of 1.0069 (95% CI: 1.0023, 1.0106) (Figure S3b).

For MDA1 O_3_, significant associations were found for the male group at the days of lag4 and lag5, with OR values of 1.0057 (95% CI: 1.0012, 1.0101) and 1.0050 (95% CI: 1.0007, 1.0093), respectively. Meanwhile, for the female group, the significant and highest association was found at the day of lag5, with an OR value of 1.0064 (95% CI: 1.0016, 1.0114) (Figure S4b).

### 3.4. Sensitivity Analysis

**Figure 6:**
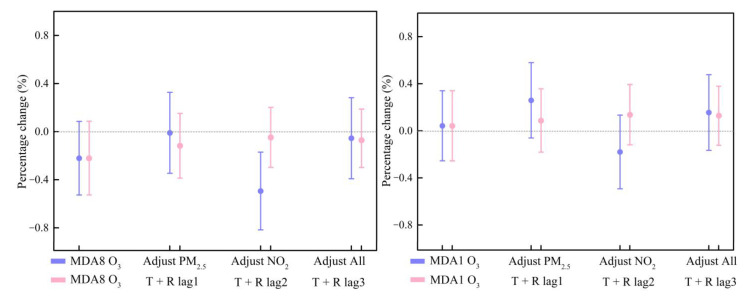
Percentage increase in daily cardiovascular hospitalization admissions with an IQR increase in MDA8 O_3_ (**left**)/MDA1 O_3_ (**right**) concentrations using the single and multiple pollutant models (purple color) and sensitivity to different lag days of meteorological factors (pink color).

Sensitivity analyses demonstrated that these results are stable by implementing the single- and multi-pollutant model. In the multi-pollutant model analysis, after PM_2.5_ was added, the OR values of different O_3_ indicators decreased slightly, but still without statistical significance (purple color in Figure 6). For the MDA8 O_3_, after adding NO_2_ or adding PM_2.5_ and NO_2_ simultaneously, the change in the effect of O_3_ on cardiovascular hospital admissions was small, with OR values of −0.0312%, −0.0015%, −0.0698%, and −0.0078%, respectively. For MDA1 O_3_, the values were 0.0049%, 0.0297%, −0.0207, and 0.0178, respectively (purple color in Figure 6).

To further verify the stability of the results, we likewise adjusted the different lag days for meteorological factors. The results showed that when the lag days of meteorological factors were 0, 1, 2, and 3 days, the OR values changed only slightly, which for MDA8 O_3_ were −0.031%, −0.017%, −0.007% and −0.008%, respectively (pink color in Figure 6). The changed OR values for the MDA1 O_3_ were 0.005%, 0.010%, 0.016%, and 0.015%, respectively (pink color in Figure 6). The very minimal change in results indicated that these results are stable.

## 4. Discussion

As far as we know, this is one of the few studies that reported the effect of different O_3_ indicators on daily cardiovascular hospital admissions. In this study, we employed a TSCC design to evaluate the short-term effect of MDA8 O_3_/MDA1 O_3_ on daily cardiovascular hospital admissions in Guangzhou from 2014 to 2018. The results showed that the associations between cardiovascular hospital admissions and O_3_ varied with different O_3_ indicators, age, gender groups, and season. The association between MDA8 O_3_/MDA1 O_3_ and daily cardiovascular hospital admissions was significantly positive in the warm season and negative in the cold season. Compared with MDA1 O_3_, MDA8 O_3_ was more strongly associated with cardiovascular hospital admissions. The effects of O_3_ on cardiovascular hospital admissions were stronger in the 60+ years age group than in the 15–60 years age group, and females were slightly more sensitive than males to O_3_. These findings not only help us understand the differences in impact of different O_3_ indicators on cardiovascular hospital admissions, but also present the differences in the effect of O_3_ on different groups.

Three O_3_ indicators (MDA8 O_3_, MDA1 O_3_, and daily 24 h average O_3_) are commonly used in O_3_-related epidemiological studies. In this study, we reviewed studies about the effects of different O_3_ indicators on cardiovascular disease worldwide since 2000 (Table 2) and found that different studies used different O_3_ indicators. For example, in the United States, daily all-cause, cardiovascular, and respiratory mortality are closely associated with MDA8 O_3_ levels [30]. Based on a generalized additive model, Qin et al. (2017) evaluated the relationship between daily O_3_ and cardiovascular mortality in 2013–2015 [31]. Their results showed that MDA8 O_3_ was positively associated with cardiovascular mortality. Gryparis et al. (2004) reported that, in Europe, a 10 μg/m^3^ increase in MDA1 O_3_ was associated with a 0.33%, 0.45%, and 1.13% increase in all-cause, cardiovascular, and respiratory mortality, respectively [32]. The daily 24 h average O_3_ indicator is also widely used in the previous epidemiological studies [17,20,33]. Sun et al. (2018) conducted an epidemiological analysis in 34 Chinese counties, using three O_3_ indicators (MDA8 O_3_, MDA1 O_3_, and daily 24 h average O_3_) from 2013 to 2015 to explore the relationship between short-term exposure to different O_3_ indicators and cardiovascular mortality [34]. Shi et al. (2020) conducted a time-series analysis in 128 Chinese counties from 2013 to 2018 and reported the associations between short-term exposure to three different O_3_ indicators and cardiovascular mortality [35].

In the aforementioned studies, we observed that different O_3_ indicators have different estimates and that the correlation between MDA8 O_3_ and cardiovascular hospital admissions was stronger than that between MDA1 O_3_ and cardiovascular hospital admissions, except in the US study [36] (Table 2). For example, Sun et al. (2018) conducted an epidemiological study in 34 Chinese counties from 2013 to 2015 considering that three different O_3_ indicators (MDA8 O_3_, MDA1 O_3_, and 24 h daily average O_3_) simultaneously to examine the associations between short-term exposure to different O_3_ indicators and cardiovascular mortality [34]. Their results showed that the association between MDA8 O_3_ and cardiovascular mortality was stronger than that between MDA1 O_3_ and cardiovascular mortality. On the basis of data from 2008 to 2009, Chen et al. (2013) used a generalized additive model to examine the effects of different O_3_ indicators on cardiovascular mortality in Suzhou [37]. Their results showed that MDA8 O_3_ and MDA1 O_3_ were positively related to daily cardiovascular hospital admissions, and the association was stronger for MDA8 O_3_ than for MDA1 O_3_. The potential reason may be that the MDA8 O_3_ indicator is the most relevant to individual exposure levels, as people come into cities for work during the daytime and return home at night. Furthermore, high O_3_ exposure during the daytime may have more negative health impacts than commute period and nighttime [38]. Li et al. (2015) also demonstrated that the health effects caused by short-term O_3_ exposure was more related to higher O_3_ concentration, such as MDA8 O_3_, rather than peak concentration, such as MDA1 O_3_ [38].

The strong solar radiation and higher temperature in summer are helpful to the photochemical reactions of NOx and VOCs, resulting in the production of the secondary pollutant O_3_. Therefore, there is a high O_3_ concentration in summer and low O_3_ concentration in winter [8,39]. According to the characteristics of O_3_, we evaluated the effects of two O_3_ indicators on daily cardiovascular hospital admissions in Guangzhou in different seasons: warm and cold. Notably, there are different associations in the warm and cold seasons, which was consistent with the previous studies. We also examined the susceptibility of different age and gender groups to O_3_ pollution during the warm season and the cold season. The results showed that, in the warm season, both MDA8 O_3_ and MDA1 O_3_ were significantly and positively related to daily cardiovascular hospital admissions. By contrast, in the cold season, the two O_3_ indicators were significantly and negatively related to daily cardiovascular hospital admissions. That is, in the warm season, there are positive associations between MDA8 O_3_/MDA1 O_3_ and daily cardiovascular hospital admissions, whereas in the cold season, there are negative associations between them, results that are consistent with other studies [2,40]. Similarly, Yang et al. (2004) aimed to use a case-crossover method to determine the association between O_3_ concentration and cardiovascular hospital admissions in Taiwan [21]. Their results showed that O_3_ was positively associated with cardiovascular hospital admissions in the warm season, as well as negatively in the cold season. Based upon O_3_ data from 2014 to 2017, Lu et al. (2022) used cardiovascular disease emergency data collected from famous hospitals in Shanghai to explore the relationship between O_3_ and cardiovascular hospital visits in different seasons [41]. Their results also showed that O_3_ had different relationships with cardiovascular hospital visits in different seasons. The underlying reason may be that temperature modulates the impacts of O_3_ on the health risks, and some studies have found that the health risks related to O_3_ exposure are enhanced in low temperature environment [42,43]. In addition, temperature has a long-term lag effect on human health, especially low temperatures, which are delayed and may last for 2–3 weeks [44]. Therefore, the modifying effects of temperature on the O_3_-cardiovascular mortality association may also result in insufficient control for temperature effects and increased O_3_ mortality risks [45].

In addition, O_3_ pollution had a lagged effect on daily cardiovascular hospital admissions. Compared to the single-day lag model, O_3_ showed similar effects on the results of the moving average lag model for different age and gender subgroups. These results suggested a cumulative effect of O_3_ pollutants on cardiovascular hospital admissions, which was similar to the previous studies [2,38,46].

There are some limitations in this study that should be mentioned. First of all, the data on air pollution in this study were collected from monitoring stations. During the study period, these may be affected by distance, climate, and other factors and could not fully reflect individual exposure, which may cause a certain impact on the estimated association between O_3_ and cardiovascular hospital admissions. Second, we did not consider some individual behavioral factors in this study, that is, we did not consider individual-level O_3_ exposure (i.e., are they smokers or not, educational background, body mass, or occupation); thus, some uncertainty may be involved. Third, our hospital admissions data include all cardiovascular and cerebrovascular diseases. The absence of detailed data about hypotension disease or the use of data that were not divided into more detailed disease subgroups may introduce some uncertainty in the results. Fourth, we do not consider the long-term trend in our model, which also may introduce some uncertainties. Finally, as our cardiovascular hospital admissions data were obtained from a large general hospital, and not all hospitals in Guangzhou, the sample size is lower than that in other epidemiological studies. Hence, care should be taken when generalizing the results to other regions.

## 5. Conclusions

This study provided evidence to examine the impact of different O_3_ indicators on cardiovascular hospital admissions in Guangzhou, China, from 2014 to 2018. To our knowledge, this is one of the few studies comparing the differences of the effects of different O_3_ indicators on cardiovascular hospital admissions. The results of this study confirmed that O_3_ pollution increases the risk of cardiovascular hospital admissions in the warm season. With the rapid economic growth of Guangzhou, there is still a long way to go in the field of controlling O_3_ pollution. Based on the above results, we have some suggestions to reduce and improve O_3_ pollution. (1) Establish a comprehensive prevention and control strategy to coordinate the control of air pollutants and greenhouse gas emissions. (2) Control the use of fossil energy, improve gas development, and promote the transformation of the energy structure. (3) Formulate goals and implementation strategies for the coordinated control of PM_2.5_ and O_3_ pollution, since PM_2.5_ and O_3_ have similar precursors. (4) Strengthen the emission control of traffic sources and coordinately control the emission of NOx and VOCs. Traffic source control is the most important strategy for O_3_ pollution control because motor vehicles contribute significantly to NOx and VOCs emissions.

## Figures and Tables

**Table 1:** Descriptive statistics of air pollutants, meteorological variables, and cardiovascular hospital admissions.

Factors	IQR	25%	50%	75%	Min	Mean	Max
COV hospital admission (number)	12	5	12	17	0	12	46
MDA1 O_3_ (μg/m^3^)	87	57	100	144	4.3	105	311
Warm period	90	77	125	167	8	125	311
Cold period	75	42	78	117	4	84	272
MDA8 O_3_ (μg/m^3^)	71	51	86	122	4	91	271
Warm period	73	70	103	143	15	111	271
Cold period	53	43	69	96	5	70	180
NO_2_ (μg/m^3^)	22	33	42	55	14	46	148
PM_2.5_ (μg/m^3^)	26	23	33	49	4	38	157
Temperature (℃)	10.6	17.1	23.9	27.7	3.3	22.2	31.7
Relative humidity (%)	13	73	80	86	28	79	97

Note: COV represents cardiovascular.

**Table 2:** Various studies on cardiovascular diseases attributed to different O_3_ indicators after the year 2000 worldwide.

References	Study Year	Study Area	Model	O_3_ Indicator	Relative Risks (*RR*)
Kazemiparkouhi et al., 2020	2000–2008	USA	LLRM	MDA8 O_3_	0.9985 (0.9975, 0.9995)
				MDA1 O_3_	1.0025 (1.0015, 1.0035)
				24 h average	0.9864 (0.9849, 0.9874)
Gryparis et al., 2004	1990–1996	23 cities, Europe	GAM	MDA8 O_3_	1.0046 (1.0022, 1.0073)
				MDA1 O_3_	1.0045 (1.0022, 1.0069)
Moshammer et al., 2013	1991–2009	Vienna, Austria	GAM	MDA8 O_3_	1.0051 (1.0026, 1.0076)
				MDA1 O_3_	1.0047 (1.0026, 1.0069)
				24 h average	1.0062 (1.0030, 1.0093)
Sun et al., 2018	2013–2015	34 counties, China	DLNM	MDA8 O_3_	1.0039 (1.0016, 1.0062)
				MDA1 O_3_	1.0031 (1.0011, 1.0051)
				24 h average	1.0066 (1.0028, 1.0104)
Yang et al., 2012	2006–2008	Suzhou, China	GAM	MDA8 O_3_	1.0074 (1.0024, 1.0122)
				MDA1 O_3_	1.0060 (1.0019, 1.0100)
				24 h average	1.0099 (1.0015, 1.0181)
Chen et al., 2013	2008–2009	Suzhou, China	GAM	MDA8 O_3_	1.0177 (1.0069, 1.0287)
				MDA1 O_3_	1.0116 (1.0025, 1.0209)
				24 h average	1.0317 (1.0124, 1.0514)
Shi et al., 2020	2013–2018	128 counties, China	GLM	MDA8 O_3_	1.0042 (1.0032, 1.0051)
				MDA1 O_3_	1.0034 (1.0027, 1.0042)
				24 h average	1.0060 (1.0046, 1.0075)

Note: M_COV_: cardiovascular mortality, LLRM: log-linear regression model, GAM: generalized additive model, GLM: generalized linear model.

## Data Availability

The data analyzed in this study are available on request from the corresponding author.

## Supplementary Materials

The following supporting information can be downloaded at: https://www.mdpi.com/article/10.3390/ijerph20032056/s1, Figure S1. Comparison the effects for an IQR increase in different O_3_ indicators (MDA8 O_3_ (a) and MDA1 O_3_ (b)) on cardiovascular hospitalization admissions in different age groups. Figure S2. Comparison the effects for an IQR increase in different O_3_ indicators (MDA8 O_3_ (a) and MDA1 O_3_ (b)) on cardiovascular hospitalization admissions in different gender groups. Figure S3. Comparison the effects for an IQR increase in MDA8 O_3_ on cardiovascular hospitalization admissions during the warm periods ((a) and (b)) and the cold periods ((c) and (d)). Figure S4. Comparison the effects for an IQR increase in MDA1 O_3_ on cardiovascular hospitalization admissions during the warm periods ((a) and (b)) and the cold periods ((c) and (d)).

## Abbreviations

**VOCs:** Volatile organic compounds; **NOx:** Nitrogen oxides; **APPCAP:** Air Pollution Prevention and Control Action Plan; **PM_2.5_:** Particulate matter with aerodynamic diameter ≤ 2.5 μg/m^3^; **MDA8 O_3_:** the maximum daily 8 h average ozone concentration; **MDA1 O_3_:** the maximum daily 1 h average ozone concentration; **CI:** Confidence interval; **TSCC:** Time-stratified case-crossover; **OR:** Odds ratio; **WHO:** World Health Organization.
