# Supplementary material for: Associations between Different Ozone Indicators and Cardiovascular Hospital Admission: A Time-Stratified Case-Crossover Analysis in Guangzhou, China
Source: Int J Environ Res Public Health. 2023 Jan 23;20(3):2056. doi: 10.3390/ijerph20032056 (PMC9916254; doi:10.3390/ijerph20032056)
Supplement: Supplementary file 1 [file ijerph-20-02056-s001.zip › ijerph-2123663-supplementary.pdf]

Supplementary Materials

Figure Captions

Figure S1. Comparison the effects for an IQR increase in different O_3 indicators (MDA8 O_3 (a) and MDA1 O_3 (b)) on cardiovascular hospitalization admissions in different age groups.
Figure S2. Comparison the effects for an IQR increase in different O_3 indicators (MDA8 O_3 (a) and MDA1 O_3 (b)) on cardiovascular hospitalization admissions in different gender groups.
Figure S3. Comparison the effects for an IQR increase in MDA8 O_3 on cardiovascular hospitalization admissions during the warm periods ((a) and (b)) and the cold periods ((c) and (d)).
Figure S4. Comparison the effects for an IQR increase in MDA1 O_3 on cardiovascular hospitalization admissions during the warm periods ((a) and (b)) and the cold periods ((c) and (d)).

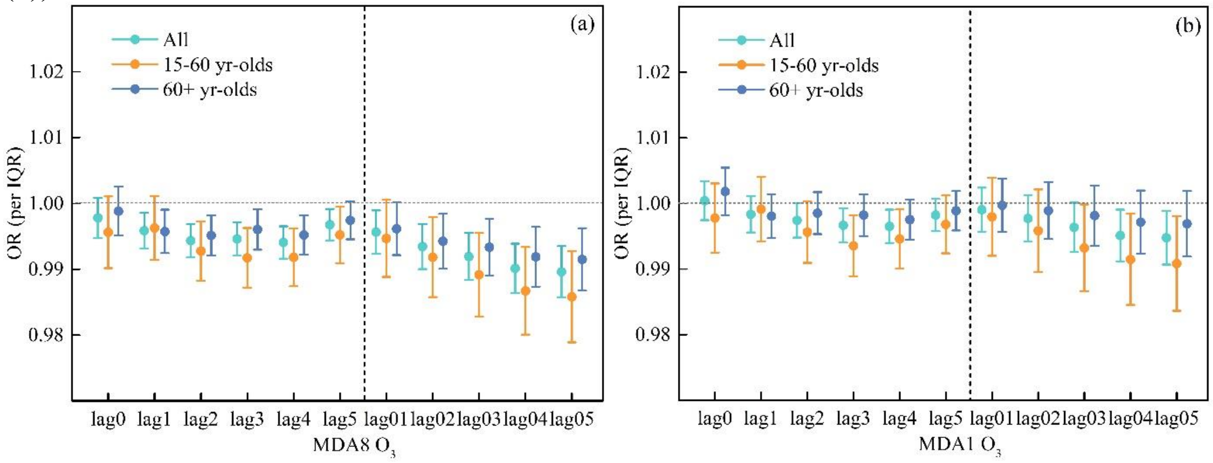

Figure S1. Comparison the effects for an IQR increase in different O_3 indicators (MDA8 O_3 (a) and MDA1 O_3 (b)) on cardiovascular hospitalization admissions in different age groups.

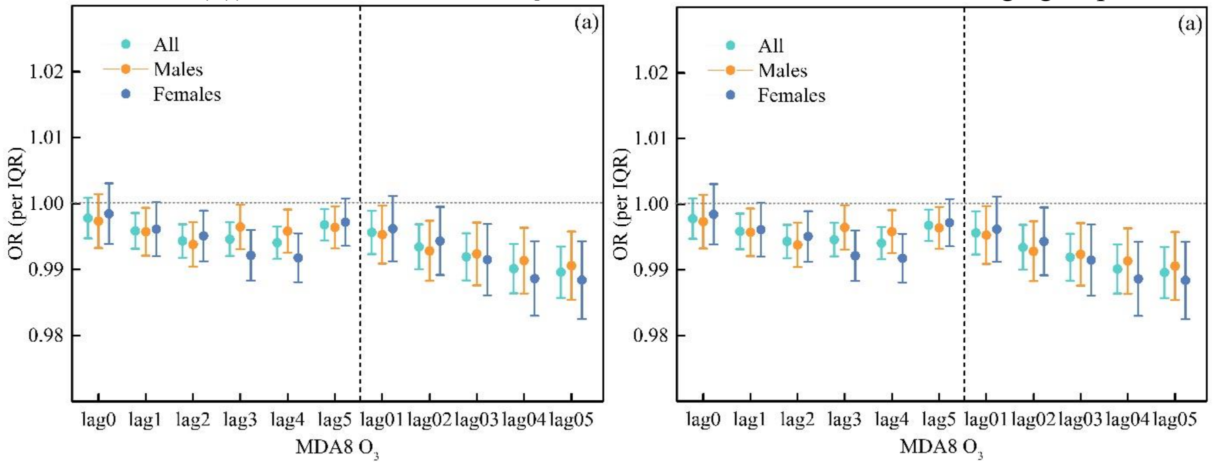

Figure S2. Comparison the effects for an IQR increase in different O_3 indicators (MDA8 O_3 (a) and MDA1 O_3 (b)) on cardiovascular hospitalization admissions in different gender groups.

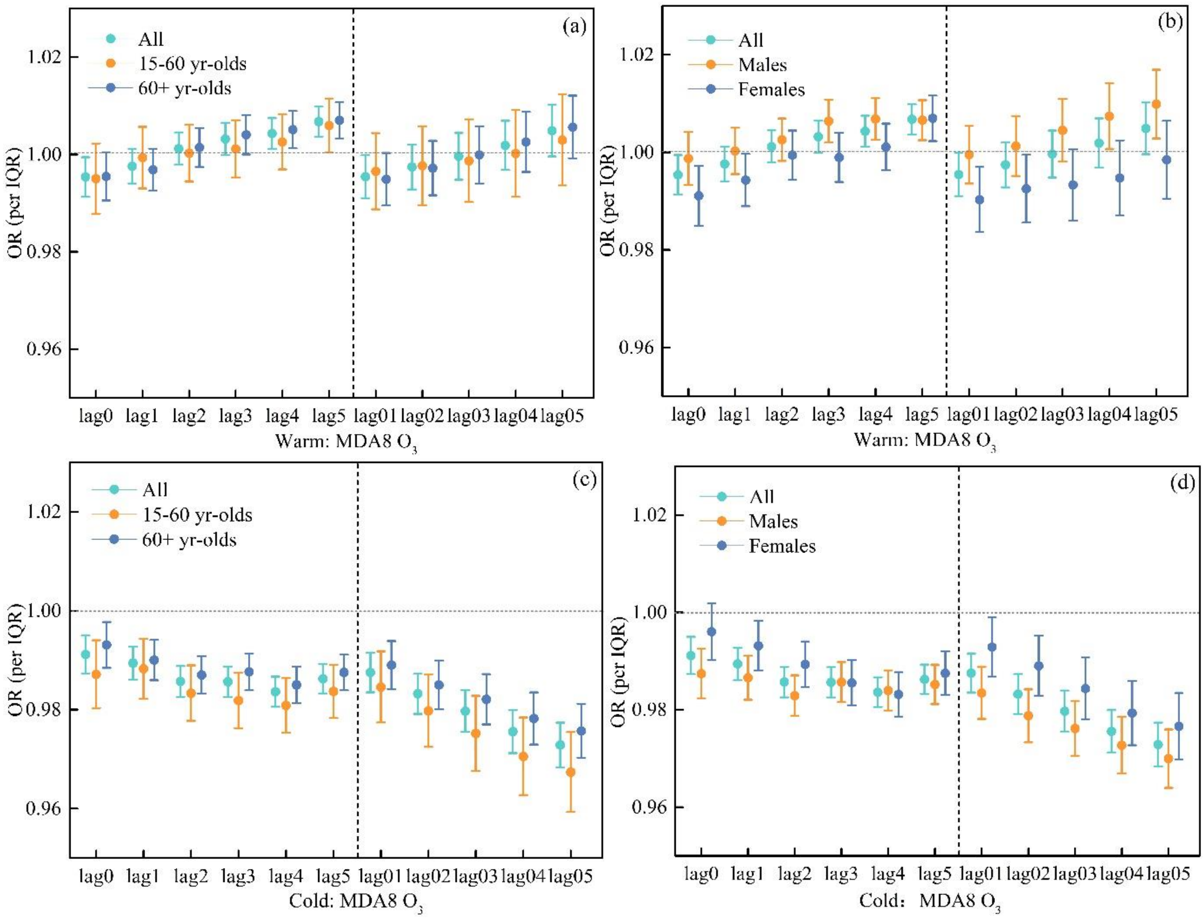

Figure S3. Comparison the effects for an IQR increase in MDA8 O_3 on cardiovascular hospitalization admissions during the warm periods ((a) and (b)) and the cold periods ((c) and (d)).

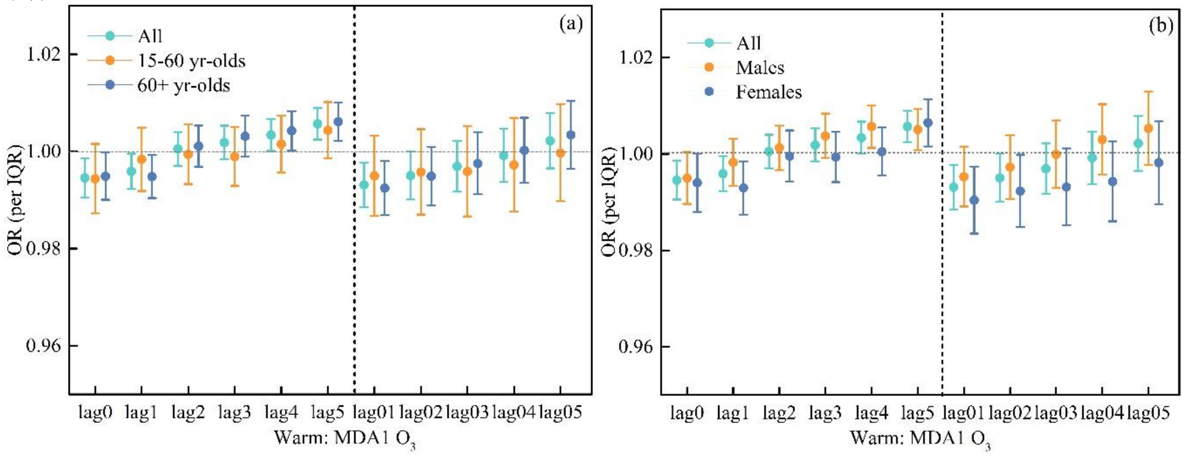

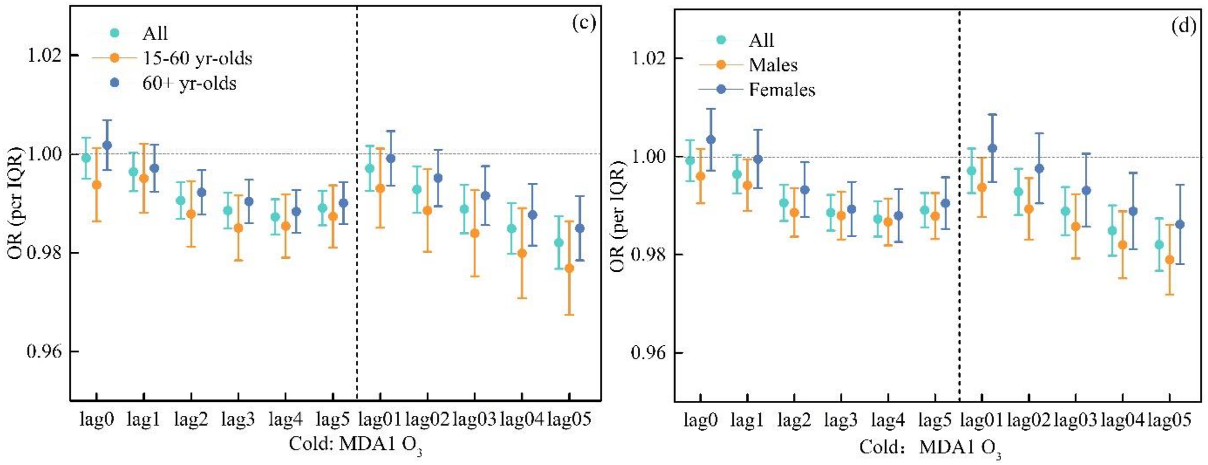

Figure S4. Comparison the effects for an IQR increase in MDA1 O_3 on cardiovascular hospitalization admissions during the warm periods ((a) and (b)) and the cold periods ((c) and (d)).
